# The Power of Lignans: Plant Compounds with Multifaceted Health-Promoting Effects

**DOI:** 10.3390/metabo15090589

**Published:** 2025-09-04

**Authors:** Marta Burgberger, Justyna Mierziak, Beata Augustyniak, Wioleta Wojtasik, Anna Kulma

**Affiliations:** Faculty of Biotechnology, University of Wrocław, Przybyszewskiego 63/77, 51-148 Wroclaw, Poland; marta.burgberger@uwr.edu.pl (M.B.); justyna.mierziak@uwr.edu.pl (J.M.); beata.augustyniak@uwr.edu.pl (B.A.); anna.kulma@uwr.edu.pl (A.K.)

**Keywords:** lignans, SDG, oxidative stress, hormonal disorders, cancer, civilization diseases

## Abstract

Lignans are plant-derived biphenolic compounds with multiple hydroxyl groups, which, upon ingestion, are metabolized by gut microbiota into enterolignans—enterolactone and enterodiol. These mammalian metabolites exhibit structural similarity to estradiol, enabling lignans to modulate hormonal balance and exert estrogen-like effects. A growing body of evidence highlights their broad spectrum of health-promoting properties, including antioxidant, anti-inflammatory, and hormone-regulating effects. Lignans have shown potential in alleviating menopausal symptoms, preventing estrogen-dependent cancers, and mitigating conditions such as cardiovascular disease, diabetes, and metabolic syndrome. Additionally, their antimicrobial activity against bacteria, fungi, and viruses is being increasingly recognized. This review provides a comprehensive and up-to-date synthesis of current knowledge. It uniquely integrates the latest insights into lignan biosynthesis, gut microbiota-mediated metabolism, and clinically relevant outcomes. Importantly, this review incorporates recent findings from prospective cohort studies and meta-analyses and sheds light on emerging therapeutic applications, including antifungal activity—an area rarely covered in earlier literature. By presenting a holistic perspective, this review advances our understanding of lignans as multifaceted compounds with significant potential in preventive and therapeutic health strategies.

## 1. Introduction

Lignans are a class of naturally occurring polyphenolic compounds present in a wide range of plant-based foods, particularly seeds (notably flaxseed), whole grains, fruits, and vegetables. Once ingested, they undergo biotransformation by the intestinal microbiota into enterolignans—primarily enterolactone and enterodiol—which closely resemble endogenous estrogens in both structure and biological activity. Due to this similarity, lignans are classified as phytoestrogens and have been the subject of increasing scientific interest for their broad spectrum of health-promoting effects.

Lignans have been shown to exert antioxidant, anti-inflammatory, and hormone-modulating actions. These properties contribute to their potential role in supporting cardiovascular and metabolic health, improving lipid metabolism, enhancing insulin sensitivity, and lowering risk factors associated with conditions such as diabetes and metabolic syndrome. Additionally, lignans may alleviate menopausal symptoms, promote bone health through their interaction with estrogen receptor β and help prevent hormone-dependent cancers such as breast and endometrial cancer by modulating estrogen signaling pathways and have neuroprotective potential.

The present review aims to provide a comprehensive overview of the biological activities of lignans, synthesizing current knowledge while also incorporating recent advances. In particular, we highlight emerging areas of research, including the neuroprotective and antimicrobial potential of lignans, and point out how interindividual differences in gut microbiota composition may influence their metabolism and effectiveness. The review also draws attention to existing knowledge gaps, such as limited clinical data and the unclear role of sex-specific responses, underscoring the need for further, well-designed human studies.

To ensure a balanced and up-to-date perspective, the literature discussed in this review was identified through a comprehensive search of databases including PubMed, Scopus, and Web of Science, using keywords such as lignans, enterolignans, phytoestrogens, health effects, and biological activity. While priority was given to studies published from 2010 onward, earlier references (1998–2006) were retained where they provided foundational mechanistic insights still relevant today.

### 1.1. Dietary Sources of Lignans

Both plant lignans and their metabolites provide numerous health benefits. As they cannot be synthesized endogenously by humans, lignans must be obtained through dietary sources. Given the broad spectrum of health-promoting effects, it is important to maintain a diet rich in lignans. The primary dietary sources of lignans include flaxseeds and sesame seeds, whole grains (such as rye, barley, and oats), and certain vegetables (e.g., broccoli, kale) and fruits (notably berries) (Table 1).

### 1.2. Biosynthesis and Bioconversion of Lignans

All phytoestrogens, including lignans, are synthesized from phenylpropanoids and simple phenols in plants [39]. Lignans are diphenolic compounds formed by the conjugation of two coniferyl alcohol residues. Stereospecific dimerization of coniferyl alcohol is catalyzed by a dirigent protein resulting in the formation of a dimeric lignan—pinoresinol, a precursor of secoisolariciresinol and matairesinol. Subsequent reactions lead to the formation of secoisolariciresinol diglucoside (SDG) and other derivatives. Sequential enantiospecific reduction in pinoresinol is carried out by reductase, generating lariciresinol followed by secoisolariciresinol (SECO). The glycosylation of SECO is catalyzed by secoisolariciresinol glucosyltransferase, which appears to be primarily localised in seeds [40]. Studies have shown that in flax seeds, SDG occurs in oligomeric form largely with lignan ester linked to 3-hydroxy-3-methylglutaric acid and glucosylated derivatives of hydroxycinnamic acids [41].

In the digestive systems of many animals, lignans ingested with food are converted by intestinal bacteria. SDG and SECO are converted mostly to enterodiol (ED) and enterolactone (EL), which are called mammalian lignans and show structural similarity to estradiol, the most active and dominant form of estrogen in the human body [13]. Their transformations are suspected to be species-specific. In the case of SDG, the gut microbiota first hydrolyzes its sugar residue, releasing SECO. Then, colonic microflora dehydroxylates and demethylates SECO, resulting in the formation of enterodiol. As for enterolactone, it can be formed by oxidation of enterodiol or directly from matairesinol. The main difference between enterolignans and plant lignans is that enterolignans have a hydroxyl group at the meta (3′) position of the aromatic ring, making them chemically stable, whereas plant lignans have oxygenated substituents at the 3′ and 4′ positions [13,40,41]. Following their formation in the gut, mammalian lignans such as enterodiol and enterolactone are absorbed in the colon and enter the hepatic portal system for conjugation in the liver. They are then excreted back into the colon through the bile duct, where they are deconjugated by the enzyme β-glucuronidase and then reabsorbed. Mammalian lignans derived from dietary phytoestrogens are present in blood, bile, faeces, urine, saliva, sperm, and milk [42,43]. Key factors affecting the metabolism of phytoestrogens include the gut microbiome composition and the overall diet, both of which influence bioavailability and conversion to active metabolites [13,44].

## 2. The Health-Promoting Properties of Lignans

**Figure 1:**
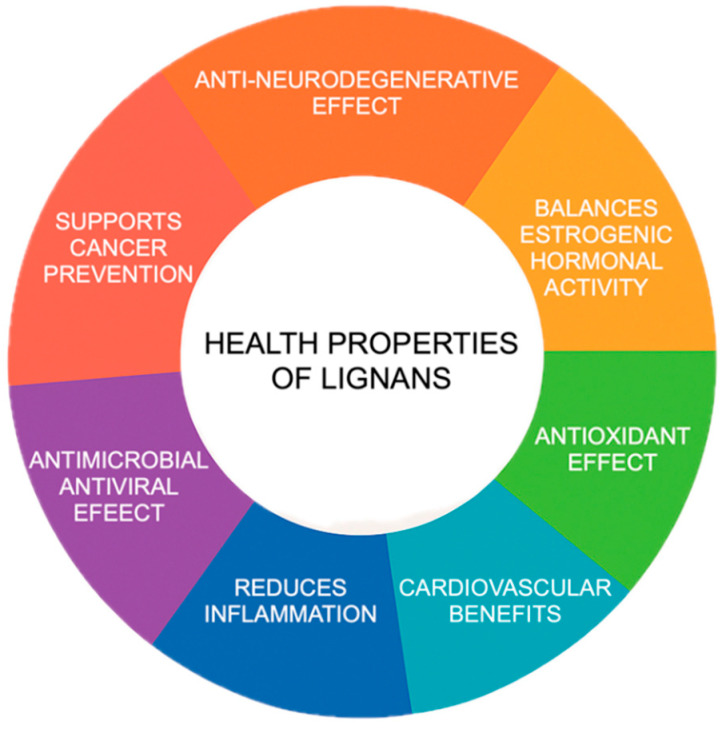
Health properties of lignans.

Lignans contribute to animal health through a range of biological effects (Figure 1). They neutralize harmful free radicals, helping to protect cells from oxidative stress and reduce inflammation. Lignans also influence hormonal pathways through their estrogen-like activity, which may affect reproduction and metabolism. Additionally, they can improve cardiovascular function by supporting healthy blood vessels and lipid profiles. Lignans aid in glucose regulation to counteract metabolic disorders and provide antimicrobial and antiviral protection. Their neuroprotective properties may also help preserve brain function, while their anti-cancer potential is linked to their ability to modulate cell growth and reduce the risk of tumor development.

### 2.1. Lignans, Oxidative Stress and Inflammation

Oxidative stress occurs when the body produces too many reactive oxygen species (ROS) compared to its antioxidant capacity. This increased level of oxidative reactions causes inflammation, which can contribute to various diseases such as hypertension [41]. Free radicals originate from various sources, including metabolic reactions, environmental pollution, ionizing radiation, poor diet, or use of stimulants. Excessive free radicals negatively affect the structure and function of the body cells, leading to dangerous diseases. Antioxidant enzymes and vitamins like C, E, and A help the body eliminate ROS more effectively. Lignans also work as antioxidants due to their structure with a high number of hydroxyl groups [40,41].

SDG, ED, and EL have been shown to effectively prevent lipid peroxidation mainly through the quenching of hydroxyl radicals, and this effect is concentration-dependent [45]. The study by Prasad, showed that lignans had higher antioxidant activity compared to vitamin E [46]. SDG and SECO are also effective antioxidants against the DPPH• radical (1,1-diphenyl-2-picrylhydrazyl radical) at concentrations of 25–200 μM, while EL and ED do not exhibit such properties against DPPH. The efficacy of lignans in controlling DNA damage induced by the peroxyl radical AAPH• (2,2′-azo-bis(2-amidinopropane)) has also been confirmed [47]. A study on sauchinone found that it can prevent iron-induced liver damage. Liver iron overload causes oxidative stress leading to hepatocyte damage and inflammation, which can result in liver fibrosis and hepatocellular cancer The action of sauchinone may depend on the activation of activated 5′AMP kinase (AMPK) dependent on LKB1 (human anti-oncogene responsible for encoding the threonine-serine kinase protein) [48].

Table 2 provides a comprehensive overview of the anti-inflammatory and antioxidant properties of lignans, particularly SDG, syringaresinol, schisandrin A, schisandrin B and honokiol. The studies compiled demonstrate that these natural compounds exert protective effects in various in vitro and in vivo models by modulating oxidative stress, inflammatory signaling pathways, and related molecular mechanisms. Syringaresinol was shown to activate Nrf2 signaling [49], inhibit pyroptosis [50], and suppress NF-κB [51] and MAPK pathways [52], resulting in the protection of tissues such as kidney, heart or lung. SchA and SchB primarily protected renal and liver tissues by reducing oxidative stress and fibrosis or ferroptosis [53,54], while honokiol showed neuroprotective effects in a model of kainic acid-induced neurodegeneration [55]. Across the studies, protective outcomes were observed in both animal models (mice or rats) and cultured cells, highlighting the therapeutic potential of these lignans in the prevention or mitigation of inflammation- and oxidative stress-related diseases.

### 2.2. Anti-Neurodegenerative Effect of Lignans

In recent decades, neurodegenerative diseases have become an increasing problem. Neurotoxic factors cause progressive damage to neurons, resulting in motor disorders, memory loss, cognitive impairment, as well as anxiety and depression. The use of lignans seems to be a good solution, as they exhibit neuroprotective properties by regulating apoptosis and modulating different signaling pathways, inhibiting expression of mRNA and inflammatory mediator proteins, and antioxidant effects [99,100,101].

Antioxidative stress, along with accumulation of amyloid beta peptide (Aβ) and tau protein aggregates in the brain and toxicity of metal ions, is one of the causes of Alzheimer’s disease (AD) [102].

Studies in rats and mice have shown that lignans from *Schisandra chinensis*, such as schisandrin A, may alleviate the neurotoxic effects of inflammation, oxidative stress, Aβ deposition, and tau protein phosphorylation. Moreover, experimental animals showed improved cognitive abilities [103]. Pinoresinol and arctigenin, present in burdock seeds, may also be helpful in the treatment of Alzheimer’s disease. These compounds have reduced memory deficits and learning and memorization problems in experimental animals. The suggested model of action of arctigenin is to reduce hyperphosphorylation of tau protein through the signaling pathway of 3-phosphatidylinositol kinase (PI3K)/Akt protein kinase/glycogen synthase kinase 3 (GSK3β). In the case of pinoresinol, the proposed mode of action is acetylcholinesterase inhibition and facilitation of calcium ion influx into neuronal cells [104,105]. Lignans isolated from *Sesame indicum* and *Acorus tatarinowii* mitigated cognitive impairment of *Drosophila melanogaster* and *Caenorhabditis elegans*. They showed protective effects against Aβ toxic aggregate [106,107,108].

Another common neurological disorder is Parkinson’s disease (PD). This disease mainly affects the musculoskeletal system, and its pathology is associated with changes in dopaminergic neurons. Studies conducted on a rat model of Parkinson’s disease suggest that sesamin may be useful in PD therapy. After administering 10–20 mg/kg/day to rats for one week, neuroprotective effects were observed. Its potential mechanism of action is through the reduction in oxidative stress, apoptosis and astrogliosis (accumulation of reactive astrocytes at the affected site). Sesamin reduced problems with motor balance in rats, decreased levels of reactive forms of oxygen and malondialdehyde (one of the markers of oxidative stress), increased the activity of superoxide dismutase (SOD), decreased the activity of some enzymes involved in apoptotic processes, and prevented damage to dopaminergic neurons [100,109]. Another compound that may be helpful in PD therapy is honokiol, obtained from the bark and seeds of *Magnolia grandiflora*. Studies using a mouse model of Parkinson’s disease showed that honokiol has both protective and therapeutic effects on damaged dopaminergic neurons and alleviates motor impairment. One potential mechanism of action of honokiol is modulation of the signaling pathway regulated by nitric oxide [110]. An increase in the number of normal dopaminergic neurons, in mice with induced Parkinson’s disease, was observed following treatment with schisandrine A. This compound also reduced the levels of inflammatory mediators and demonstrated antioxidant activity. In addition, schisandrine A activated proteins associated with autophagy [111]. Giuliano et al. reported that treatment with 7-hydroxymatairesinol was able to slow the progression of dopaminergic neuronal terminal degeneration in the rat PD model. While this treatment did not fully protect dopaminergic cell bodies, it shows potential for alleviating symptoms of Parkinson’s disease [112].

Lignans represent a promising group of compounds with neuroprotective potential, supported by extensive experimental evidence. Specific findings, disease models, lignan types, and molecular targets are summarized in Table 3. This compilation presents an overview of current research on the neuroprotective effects of selected lignans in neurodegenerative diseases, with a focus on Alzheimer’s disease (AD) and Parkinson’s disease. Numerous studies demonstrate that lignans exert multifaceted neuroprotective effects: they reduce oxidative stress, inhibit β-amyloid aggregation, modulate mitochondrial autophagy, improve mitochondrial function, and attenuate neuroinflammatory processes via signaling pathways such as NF-κB, ERK/MAPK, and SIRT3 [66,113,114,115]. In PD models, schisandrin A and 7-hydroxymatairesinol protected dopaminergic neurons and improved motor function [111,112]. In ALS models, honokiol enhanced mitochondrial dynamics and antioxidant capacity, prolonging survival in transgenic mice [116].

### 2.3. Lignans and Osteoporosis

Osteoporosis is a global health problem that affects many older people, especially postmenopausal women, for whom estrogen deficiency is a risk factor for developing the disease [137,138]. Estrogens inhibit bone resorption and block the production of IL-1, IL-6, and TNF-α, which stimulate osteoclasts. Estrogens also stimulate the synthesis of bone matrix components, collagen, and non-collagen proteins. The protective abilities of plant estrogens against bone density loss are most likely due to their affinity for β estrogen receptors, which enables them to inhibit osteoclasts and activate osteoblasts [139,140,141].

A large number of postmenopausal women consume flax seeds as a supplement to their pharmacological drugs. Studies conducted on ovariectomized female rats suggest that lignans, including those from flax, may be useful in protecting against bone fractures or lumbar vertebrae bone loss [142,143,144]. Studies have shown that sezamine can induce osteoblast differentiation by activating the p38 and ERK/MAPK signaling pathway. What is more, it may indirectly influence osteoclast development by enhancing the expression of OPG (osteoprotegerin) and suppressing the expression of RANKL (Receptor Activator for NF-κB Ligand) [141]. It has also been shown that the anti-osteoporotic activity of matairezinol is derived from its ability to counteract osteoclastogenesis Via the p38/ERK-NFATc1 signaling pathway [145].

Table 4 summarizes recent research on specific lignans—such as pinoresinol diglucoside, sesamin, arctigenin, and arctin—highlighting their therapeutic potential in osteoporosis and osteoporotic fracture healing. These compounds were shown to enhance osteogenesis, suppress osteoclast activity, and improve bone structure through modulation of signaling pathways like PI3K/Akt, NF-κB, and Wnt/β-catenin [146,147]. Both in vitro and in vivo studies, suggest their promise as natural treatments for osteoporosis.

### 2.4. Lignans and Cardiovascular Diseases

The leading cause of death and disability worldwide are cardiovascular diseases (CVDs), with myocardial infarction and stroke being the predominant causes of disability. Various factors such as hypertension, obesity, inflammation, atherosclerosis, oxidative stress, diabetes, and dyslipidemia promote the development of CVDs. Women are particularly susceptible to CVDs during the postmenopausal period due to the decrease in the concentration of endogenous estrogens, which have a beneficial effect on lipid metabolism, coronary vessels dilation, insulin sensitivity, and blood coagulation. Additionally, estrogens regulate endogenous lipid synthesis by reducing the total cholesterol concentration by approximately 9% and triglyceride (TAG) concentration by approximately 10% [41,158,159]. The development of atherosclerosis is influenced by free radicals and hypercholesterolemia, characterized by high levels of LDL (low density lipoprotein) cholesterol and low levels of HDL (high density lipoprotein) cholesterol.

Lignans exert protective effects both directly, as antioxidants, and indirectly, through the activation of estrogen receptors. SDG, as an antioxidant, has been shown to reduce the development of atherosclerosis caused by high levels of LDL cholesterol. A lignan complex isolated from flax seeds has been shown to reduce the development of atherosclerosis by 34.37%, supporting its potential role in preventing atherosclerosis and lowering the risk of coronary artery disease and stroke. In hypercholesterolemic rabbits, treatment with flaxseed lignans resulted in a 20% reduction in total cholesterol, a 14% decrease in LDL cholesterol, a 34% reduction in the total cholesterol to HDL cholesterol ratio, and a 35% decrease in malondialdehyde (MDA), a marker of lipid peroxidation. Additionally, HDL cholesterol levels rose by 30% in hypercholesterolemic rabbits and by 25% in healthy ones treated with the lignan complex. In contrast, the treatment did not significantly alter total cholesterol, LDL cholesterol, or MDA levels in healthy rabbits. These results indicate that flaxseed lignans may offer cardiovascular benefits primarily in conditions associated with high cholesterol, helping to prevent the progression of atherosclerosis [160].

What is more, studies demonstrate that natural compounds such as flaxseed, arctigenin, schisandrin B, and sesamine exhibit strong cardioprotective properties. These include antiarrhythmic effects, reduced infarction size, and attenuation of oxidative stress and inflammation, making them promising agents in the prevention and treatment of cardiovascular diseases [108,161,162,163,164]. A summary of the cardioprotective mechanisms of lignans is presented in Table 5. Studies in animal models have shown that a flaxseed-rich diet, as well as supplementation with its key components—alpha-linolenic acid (ALA) and SDG—can significantly reduce the incidence of arrhythmias, minimize infarct size, limit left ventricular dilatation, and lower levels of the pro-inflammatory cytokine TNF-α. These results suggest that flaxseed may support both prevention and treatment of arrhythmias and facilitate cardiac repair after myocardial infarction [164].

Myocardial ischemia/reperfusion (MI/R) injury and acute myocardial infarction are associated with oxidative stress, inflammation, and arrhythmias. Arctigenin (ATG) has shown significant protective effects in animal models of these conditions. In rats pre-treated with ATG before MI/R, the frequency and duration of ventricular arrhythmias—including fibrillation and tachycardia—were markedly reduced, along with a decrease in infarct size. These effects were accompanied by increased antioxidant enzyme activity and a reduction in malondialdehyde (MDA) levels, indicating lowered oxidative stress. The antiarrhythmic and cardioprotective effects of ATG are likely mediated by activation of the Nrf2 signaling pathway, as well as modulation of iNOS, COX-2, ERK1/2, and HO pathways. These findings suggest that ATG could be a promising agent in preventing infarction and limiting damage caused by ischemia–reperfusion injury [108,163].

Myocardial infarction causes significant damage to heart tissue through inflammation and apoptosis of cardiac cells. Animal studies have shown that both schisandrin B (Sch B) and sesamine exert cardioprotective effects following infarction. Schisandrin B reduces infarct size and apoptosis by activating the PI3K/Akt signaling pathway, increasing phosphorylated Akt levels, and decreasing the expression of pro-apoptotic markers such as caspase-3 and the Bax/Bcl-2 ratio. Sesamine, on the other hand, mitigates myocardial damage by reducing cardiac cell apoptosis and suppressing inflammation. Its effects are linked to the inhibition of the NF-κB pathway and decreased cytokine expression. Together, these compounds demonstrate strong protective effects on the heart after infarction, supporting their potential role in limiting cardiac injury and improving outcomes following myocardial events [161,162].

Human studies have demonstrated the health benefits of SDG supplementation in cardiovascular diseases. The effects of different SDG doses on total cholesterol, LDL cholesterol, metabolic syndrome, and glucose concentration were studied. Zhang et al. have shown that a dose of 600 mg SDG per day effectively reduces total cholesterol, LDL cholesterol, and fasting glucose in plasma in patients with hypercholesterolemia [204]. In the case of moderate hypercholesterolemia in men, consuming 100 mg SDG per day is enough to effectively lower cholesterol, and such supplementation reduces the risk of liver disease. This is related to a reduced ratio of LDL cholesterol to HDL cholesterol and a decrease in glutamine pyruvate transaminase and γ-glutamyl transpeptidase [205]. Studies on the role of flaxseed supplementation among postmenopausal women show that such dietary supplementation improves the lipid profile, which may have a beneficial effect on the cardiovascular system [206]. Studies on middle-aged Finnish men by Vanharanta et al. have shown a significant association between increased serum enterolactone and a reduced risk of death from ischemic heart disease or cardiovascular disease [207]. One of the key processes in ischemic heart disease is the apoptosis of myocardial cells, mediated by oxidative stress. Studies on rat cardiomyocytes have shown that SDG is able to reduce the adverse effect of H_2_O_2_ and act as an anti-apoptotic compound by activating the JAK2/STAT3 signaling pathway [170].

Hypertension is an important risk factor for cardiovascular disease, and diet plays an essential role in its prevention and control. Epidemiological studies have suggested an inverse relationship between polyphenol intake and cardiovascular disease. Clinical studies have confirmed that daily oral supplementation of the lignan complex is significantly associated with plasma concentrations of linseed lignans (free and conjugated forms): secoisolariciresinol, enterodiol (ED), and enterolactone (EL). Participants supplementing with the lignan complex (600 mg SDG/day for 6 months) showed a significant decrease in systolic blood pressure from a mean of 155 ± 13 mm Hg at baseline to 140 ± 11 mm Hg at 24 weeks. These data suggest a relatively safe ability to lower systolic blood pressure, which is an important risk factor for cardiovascular disease [208]. Conversely, a study on the relationship between estimated phytoestrogen intake and hypertension, conducted among 1936 men and women aged at least 18 years, based on dietary forms maintained by the subjects, found a significant association between the intake of pinoresinol and blood pressure reduction among men [209].

It is worth mentioning podophyllotoxin (PPT), a lignan that exhibits strong cardiotoxicity, as demonstrated in in vivo studies by elevated cardiac injury markers and histopathological changes. Its toxic mechanism involves oxidative stress (mediated by CYP2E1), a pronounced inflammatory response driven by arachidonic acid metabolites, and disruptions in cardiomyocyte energy metabolism. Additionally, PPT activates the SIRT1/PPAR/NF-κB and Akt1/SREBP 1c signaling pathways, further exacerbating cardiac damage. Studies suggest that PUFA supplementation may offer partial protection against these effects, opening potential avenues for mitigating PPT-induced toxicity [210,211,212].

### 2.5. Lignans, Diabetes and Metabolic Syndrome

Metabolic syndrome (MS) increases the risk of developing type 2 diabetes and cardiovascular diseases associated with atherosclerosis, making it a major contributing factor in their pathogenesis. MS is characterized by a cluster of factors, including insulin resistance, hyperinsulinemia, abdominal obesity, impaired glucose tolerance, microalbuminuria, hypertriglyceridemia, decreased HDL cholesterol, hypertension, and pro-inflammatory and pro-thrombotic conditions [213,214]. An example of a lignan that significantly inhibits metabolic syndrome is honokiol, an active ingredient found in the traditional Chinese herb magnolia. This was evidenced by improvements in hepatic steatosis, liver fibrosis, adipose tissue inflammation, and insulin resistance. The effect of honokiol was primarily due to AMPK activation. It directly bound to the AMPKγ1 subunit to potently activate AMPK signaling [215].

Diabetes is a group of metabolic diseases characterized by chronic hyperglycemia, which contributes to diabetic retinopathy, diabetic nephropathy, peripheral and autonomic neuropathy, ischemic heart disease, and hypertension. The main cause of diabetes is either a defect in insulin production by the β-cells of the pancreas (type 1) or impaired insulin signaling/action (type 2). Insulin resistance defined as reduced sensitivity of tissues to insulin, is the main cause of type 2 diabetes, which can later lead to impaired secretory function of pancreatic islet β cells [216,217].

The lignan complex extracted from flax has been shown to improve glycemic control. Studies by Prasad et al. in female rats have demonstrated that diabetes is correlated with increased oxidative stress, total cholesterol, triacylglycerols (TAG), and glycated hemoglobin A1C. No elevation of any of these parameters was observed in non-diabetic rats. Animal models with a predisposition to diabetes have shown that treatment with SDG reduces the incidence of diabetes and delays its development. This effect was associated with a reduction in oxidative stress (decrease in MDA and glycated hemoglobin). It has also been observed that taking 40 mg SDG per kilogram of body weight per day effectively delays the onset of type 2 diabetes [218,219].

Human studies have also demonstrated the beneficial effects of SDG against type 2 diabetes. In a study conducted by Pan et al. on individuals aged 50 to 79 years, it was observed that the participants who included lignans in their diet experienced a significant reduction in their HbA1C (glycated hemoglobin) levels as compared to those in the placebo group. The lower the concentration of glycated hemoglobin HbA1C, the lower the risk of developing complications associated with diabetes [220]. In a follow-up study conducted, one year later, by the same group on patients with type 2 diabetes (26 men and 44 postmenopausal women) who had elevated C-reactive protein (CRP) levels—a marker of inflammation— it was found that the increase in CRP was lower in women supplementing with SDG compared to the placebo group. However, no such relationship was observed in men [221]. The results of studies on the effect of flax lignans during exercise training on metabolic syndrome and the risk of osteoporosis in individuals aged 50 years or older have shown that flax lignans reduce diastolic blood pressure and triacylglycerols, as well as parameters of six metabolic syndrome risk factors (fasting glucose, HDL cholesterol, TAG, abdominal obesity, blood pressure, and inflammatory cytokines). These decreases were observed only in men, and no such changes were observed in women [222].

Examples of molecular mechanisms of action of other selected lignans in managing and preventing diabetes are presented in Table 6. Sesamin is not included in the table because its role as bioactive compounds with antioxidant, anti-inflammatory, anti-hypertensive, anti-infective, anti-obesity, anti-diabetic, anti-thrombotic, and lipid-lowering effects has recently been summarized in detail in several reviews [223,224,225,226].

### 2.6. Lignans and Breast Cancer

The most common cancer among women is breast cancer [243]. Breast cancer is characterized by significant heterogeneity. Based on immunohistochemical expression of hormone receptors (ER), progesterone receptor (PR), and human epidermal growth factor receptor (HER2), it is classified into distinct subtypes. Four subtypes of breast cancer are commonly distinguished: luminal A (ER+, PR+, HER2−), luminal B (ER+, PR+/PR−, HER2+/HER2−), HER2-positive (ER−, PR−, HER2+), and triple-negative (ER−, PR−, HER2−). Breast cancer develops through distinct molecular mechanisms depending on its subtype. Luminal breast cancers are driven by estrogen signaling through ERα, which regulates genes involved in cell proliferation, while dysregulation of this pathway—particularly Via PI3K/AKT/mTOR activation due to PIK3CA mutations and PTEN loss—promotes tumor growth. In contrast, HER2-positive breast cancer is characterized by HER2 gene amplification, which triggers hyperactivation of PI3K/AKT and MAPK pathways, leading to aggressive cell proliferation. Triple-negative breast cancer (TNBC), lacking hormone receptors and HER2, is primarily linked to BRCA1/BRCA2 and TP53 mutations, which impair DNA repair and promote genomic instability, making it more aggressive and genetically heterogeneous [244]. Lignans may reduce breast cancer risk through multiple molecular mechanisms. They modulate estrogen receptors, inhibit aromatase, and reduce estrogen-driven cell proliferation. They also have antioxidant and anti-inflammatory effects, and can influence gene expression related to cell cycle control and DNA repair [245]. Lignans not only inhibit the development of breast cancer but also all estrogen-dependent cancers (ovarian, and endometrial cancers) by lowering estrogen levels and other enzymes involved in steroid hormone synthesis. These compounds are antagonistic to estrogens, thereby inhibiting the growth of cancer cells [13,246]. Examples of the mechanism of action of lignans on different breast cancer subtypes are presented in Table 7. A meta-analysis from 2021 found that higher ligan intake was correlated with better survival for breast cancer patients [247], including postmenopausal patients [248]. The risk of breast cancer is also significantly reduced in women consuming higher amounts of lignans [249,250].

Consumption of lignans during mammary gland development can have protective effect later in life. It is believed that the reduction in highly proliferating structures called terminal ending buds (TEBs) in the developing gland, by differentiation into structures called alveolar buds (Abs), leads to a reduced risk of developing mammary gland cancer due to the lower proliferation of AB structures than TEB. During the early development of the mammary glands, increasing levels of endogenous estrogen promote the branching of the milk duct, which ends in TEB structures. AB structures are less proliferative than TEB structures, so they are potentially less susceptible to carcinogens. Animal model studies have confirmed that feeding rats during pregnancy and lactation with flax seeds has a positive effect on the differentiation of the mammary gland [251]. In addition, the metabolites of flax seeds or SDG itself, taken together with the mother’s milk at such an early stage of development of the mammary gland in the offspring, affect the reduction in susceptibility to its carcinogenesis later in life [252]. SDG may affect mammary gland morphogenesis by modulating EGFR (epidermal growth factor receptor) and ER (estrogen receptor) signaling pathways [253]. In addition, when cancer is already present, dietary intake of flaxseed or SDG alone reduces the size and number of mammary gland tumors, as well as the invasiveness and malignancy of the cancer [254].

**Table 7:** Mechanism of action of lignans on different subtypes of breast cancer.

Lignans	Type of Breast Cancer	Action	Mechanism	**Reference**
Trans-(±)-kusunokinin	triple-negative	attenuation of breast cancer cell migration	inhibition of AKR1B1 enzyme activity resulted in the protection of glucose-induced cellular oxidation	[255]
(−)-kusunokinin	luminal A	inhibited of breast cancer cell (MCF-7) migration, proliferation, cell cycle and metastasis	decrease in cell proliferation (c-Src, PI3K, Akt, p-Erk1/2 and c-Myc), cell cycle (E2f-1, cyclin B1 and CDK1) and metastasis (E-cadherin, MMP-2 and MMP-9) proteins	[256]
Matairesinol	triple-negative	induction of apoptosis	reduction in the viability of M2a and M2d macrophages and repolarization them to M1 phenotype	[257]
Secoisolariciresinol diglucoside (SDG)	luminal A	reducing tumor cell proliferation	reduction in PS2, BCL2, and IGF-1R ERα, ERβ, EGFR, BCL2 mRNA expression and PMAPK protein	[258]
		inhibition of cell proliferation, induction of apoptosis	decreased mRNA expressions of Bcl2, cyclin D1, pS2, ERα, and ERβ, epidermal growth factor receptor, and insulin-like growth factor receptor; decreased phospho-specific mitogen-activated protein kinase expression	[259]
	triple-negative	reduction in tumor growth	inhibition of NF-κB activity	[260]
SDG derivatives	luminal A	induction of apoptosis; reduction in proliferation	the cleavage of PARP inhibition of ERα	[261]
		induction of apoptosis	overexpressed pro-apoptotic genes (*TP53*, *CDKN1A*, and *BAX*) and underexpressed anti-apoptotic genes (*BCL-2*)	[262]
	luminal A, triple-negative	cytotoxic, anti-proliferative and pro-oxidant activity	reduction in intracellular oxidative stress and DNA damage	[263]
Podophyllotoxin	triple-negative	inhibition of cell proliferation, migration and invasion; regulation of cell cycle and induction of apoptosis	inhibition of CDC20, PLK1 expression, and CDK1 and increase the expression of P53	[264]
Lariciresinol	HER2-positive	induction of apoptosis	overexpressed pro-apoptotic genes (*TP53*, *CDKN1A*, and *BAX*) and underexpressed anti-apoptotic genes (*BCL-2*)	[262]
Sauchinone	triple-negative	attenuation of proliferation, migration, and invasion	suppresion of Akt-CREB-MMP13 signaling pathway	[265]
	HER2-positive	inhibition of progression;	regulation of miR-148a-3p/HER-2 axis; increased miR-148a-3p expression, so downregulated HER-2 expression	[23]
Sesamin	HER2-positive	inhibition of cell proliferation; inducing cell cycle arrest; induction of apoptosis	increasing of P53 and Chk2; activation of the Bax and caspase-3 pathways	[266]
	triple-negative	suppression of proliferation and migration	decreases the expression of PD-L1 Via the downregulation of AKT, NF-κB, and JAK/Stat signaling	[267]
Schisandrin B	triple-negative	induction of cell cycle arrest and apoptosis, inhibition of migration and colony formation of tumor cells	suppression of signal transducer and activator of transcription-3 (STAT3) phosphorylation and nuclear translocation	[268]
		suppression the growth, migration, and invasion	inhibits interleukin (IL)-1β production of TNBC cells, hindering its progression	[269]
Schisandrin A	triple-negative	inhibition of migration and induction of apoptosis	reduction in the activation of EGFR, PIK3R1, and MMP9 and increases the expression of cleaved-caspase 3,	[270]
		induction of cell cycle arrest and apoptosis	regulation of the Wnt/ER stress signaling pathway	[271]
Schisandrol A	luminal A	promotion of proliferation	activation of ERK, PI3K, Akt, and Erα	[272]
Arctigenin	triple-negative	inhibition of the metastasis	inhibition of the activity of matrix metalloproteases MMP-2, MMP-9 and heparanase	[273]
		reduction in proliferation and induction of apoptosis	inhibition of binding of STAT3 to genomic DNA	[274]
	luminal A, triple-negative	exhibition of anti-metastatic activity	inhibition of MMP-9 (extracellular matrix metalloproteinase) and uPA (plasminogen ukinase activator) Via Akt, NF-κB and MAPK signaling pathways, regardless of estrogen receptor expression	[275]
Honokiol	luminal A, luminal B, triple-negative, HER2-positive	inhibition of growth associated with a G1-phase cell cycle arrest and induction of caspase-dependent apoptosis	attenuate the PI3K/Akt/mTOR (Phosphoinositide 3-kinases/Akt/mammalian target of rapamycin) signalling by down-regulation of Akt phosphorylation and upregulation of PTEN (Phosphatase and Tensin homolog deleted on chromosome Ten) expression	[276]
	triple-negative	inhibition of proliferation, suppression of migration and induction of apoptosis	modulating the miR-148a-5p-CYP1B1 Axis	[277]
	luminal A	induction of apoptosis influence on the cell cycle	suppression of the expression of Bcl-2 decreases the cyclin D1 expression	[278]

### 2.7. Lignans and Menopause

Menopause is the cessation of menstruation, caused by the loss of follicular activity of the ovaries, after which no bleeding has occurred for 12 consecutive months. Symptoms of decreased estrogen secretion include abnormal bleeding from the birth canal, hot flashes with sweating, palpitations, and depressive symptoms. These symptoms can last from six months to several years and occur in 65% of women. In addition, there may be an increased risk of cardiovascular disease and cancer (especially breast cancer). One of the ways to prevent the symptoms of menopause is Hormone Replacement Therapy, which involves the administration of replacement doses of female sex hormones. However, there are many contraindications to its use, including diabetes, osteoporosis, estrogen-dependent cancers, and thromboembolic disease. An alternative to such therapy is the use of phytoestrogens [279,280]. Studies have also confirmed that the use of tablets containing a mixture of isoflavones, lignans, and *Cimicifuga racemosa* (black cohosh) for a minimum of 3 months effectively lowers the Kupperman Index, which is used to assess menopausal symptoms. The combination of those components shows a reduction in postmenopausal symptoms within 24 h [279,281]. A clinical study published in 2025 confirmed the effectiveness of this combination in alleviating menopausal symptoms compared to a placebo confirming its potential as an effective therapeutic option. Clear results were observed—scores on the Menopause Rating Scale decreased by 48%, with significant improvements across all domains (somatic, psychological, and urogenital). Additionally, a 6.7% reduction in FSH levels and a 12.6% increase in estrogen concentrations were noted. Adverse effects were minimal [282].

A meta-analysis by Touillaud et al. (2009) showed that in postmenopausal women, high levels of lignan intake may be associated with a reduced risk of breast cancer [283]. Cardiovascular disease is less common in premenopausal women than in men, but estrogen deficiency increases its incidence [284]. However, data show that higher long-term lignan intake is significantly associated with a reduced risk of total coronary heart disease (CHD) in both men and women [285]. Estrogen activates its receptors, ERα and ERβ, which stimulate endothelial nitric oxide synthase (eNOS) and promote nitric oxide (NO) production, leading to vascular relaxation. It also improves lipid profiles by increasing HDL and lowering LDL levels. In contrast, estrogen deficiency can result in elevated LDL, triggering chronic inflammation in the aorta and liver through macrophage activation and cytokine release (e.g., TNF-α, IL-1β, PAF-AH), ultimately contributing to hypercholesterolemia and atherosclerosis [286,287].

Most symptoms of menopause are associated with chronic inflammation, as estrogen deficiency often results in a marked increase in pro-inflammatory cytokine levels in the serum, liver, bone, and brain. Activation of the NF-κB pathway and the resulting increase in cytokine production across various organs may contribute to the development of atherosclerosis, osteoporosis, and psychological disorders such as depression. Activation of the NF-κB pathway and the resulting in-crease in cytokine production across various organs may contribute to the development of atherosclerosis, osteoporosis, and psychological disorders [288,289,290].

In menopausal women, estrogen deficiency causes substantial and lasting bone loss, primarily due to the impaired regulation of osteoclast activity. Sesamin appears to be the most promising lignan as a therapeutic agent for postmenopausal osteoporosis. Sesamin promotes bone formation through upregulation of Wnt/β-catenin signaling, while concurrently inhibiting bone resorption by downregulating the NF-κB pathway. Moreover, DANCR has been identified as a central regulator of sesamin-induced modulation of bone formation and resorption [146]. A study in rats demonstrated that daily supplementation with sesame oil can provide osteoprotective effects in osteoporotic rats by increasing aromatase and estradiol levels, as well as by modulating the imbalance between bone formation and resorption [291]. Another lignan with anti-osteoporotic activity is matairesinol, which may exert its effects through anti-osteoclastogenic mechanisms involving the p38/ERK-NFATc1 signaling pathway [145].

In a model of osteoporotic bone fracture, sesamin was shown to significantly enhance callus formation and increase cartilage area during the early healing phase, as well as to reduce fracture gap and increase callus volume during the late phase of femoral fracture healing in OVX mice, indicating the therapeutic potential of this lignan in the treatment of osteoporotic fracture [155]. However, an earlier study conducted also in a rat model showed that administration of methanolic extracts from sesame seeds led to a decrease in bone mass, as indicated by bone densitometry and bone formation markers. This highlights the need for further research to clarify the role of sesamin and sesame seeds in mitigating bone loss observed in postmenopausal women [292].

Secoisolariciresinol (SECO) has been found to have a positive effect on reducing depression, which is one of the symptoms of menopausal syndrome. A dose of 10 mg/kg SECO can counteract depression-like behaviors and probably Via an enhancing effect on norepinephrine and dopamine levels [293]. In mice exposed to stressors for six weeks, daily administration of 50 mg/kg of sesamin increased 5-HT levels and decreased norepinephrine levels in the striatum. Sesamin significantly alleviated memory impairment and depression-like behaviors induced by chronic mild stress by inhibiting neuroinflammation through the suppression of excessive microglial activation and the expression of inflammatory mediators, including iNOS, COX-2, TNF-α, and IL-1β, in the hippocampus and cerebral cortex [294]. Furthermore, lignan-rich extracts from Schisandra chinensis and Kava-Kava have also been suggested to alleviate depressive symptoms [295,296].

Estrogen deficiency can also cause urinary incontinence because estrogen receptors are present in the genitourinary tract and pelvic floor musculoskeletal structures. Increased levels of ED and EL (derived from lignans) in urine have been found to decrease the likelihood of acute and mixed urinary incontinence, as demonstrated by studies conducted among postmenopausal women [297]. A study by Hallund et al. conducted among postmenopausal women found that the lignan complex isolated from flax reduces the concentration of C-reactive protein (CRP), which is not only a marker of inflammation but also a marker for assessing the risk of vascular disease, heart disease, or stroke (its value then increases significantly) [298,299]. In 2024, a study involving 51 women confirmed the beneficial effects of flax lignans on cardiometabolic risk after menopause. Participants consumed 40 g of flaxseed daily for 8 weeks, which led to an improved lipid profile (increased HDL and decreased LDL) and a significant reduction in CRP levels [300].

In summary, lignans may represent a promising compound with broad preventive and therapeutic potential, particularly in addressing health challenges associated with menopause, such as obesity and cardiovascular diseases

### 2.8. Antimicrobial and Antiviral Properties of Lignans

There are many emerging drug-resistant microbes, making it crucial to find alternative compounds with antimicrobial properties. Besides their already mentioned activities lignans have been shown to possess antiviral, antibacterial, and antifungal properties.

Lignans display broad antimicrobial activity. For example, nortrachelogenin disrupts bacterial membranes and is effective against antibiotic-resistant strains [301]. Hinokinin targets *Staphylococcus aureus* and MRSA [302]. Sesamin inhibits L-tryptophan indole-lyase, reducing production of indoxyl sulfate, a toxin linked to kidney disease [303]. Flax-derived lignans, including SDG, inhibit growth of *S. aureus*, *S. aureus*, *E. coli*, *P. aeruginosa* and *B. subtilis* [304,305]. 7-Hydroxymatairesinol (HMR) and lignans from Sonchus asper also act against various pathogens, including Klebsiella, Proteus, *S. cerevisiae*, and *Staphylococcus* spp. [306,307]. One of the key antibacterial mechanisms of lignans is the disorganization or disruption of the bacterial plasma membrane, destabilizing membrane potential, impairing substance transport and in some cases leading to loss of membrane integrity, leakage of intracellular contents, and ultimately cell death. Lignans have been shown to inhibit bacterial biofilm formation by interfering with cell adhesion. Additionally, some lignans act as inhibitors of essential bacterial enzymes, such as L-tryptophan indole-lyase (TIL). TIL plays a significant role in bacterial physiology, influencing biofilm formation, antibiotic resistance, plasmid retention, and virulence. Inhibiting this enzyme with lignans may therefore reduce bacterial pathogenicity and survival. Moreover, lignans can promote oxidative stress in microbial cells and inhibit nucleic acid synthesis, further contributing to their antimicrobial activity [301,303,308,309,310]. Lignans represent a large class of natural compounds with broad antiviral properties or example, lignans isolated from *Boesenbergia thorelii* roots have shown promising antiviral activity against HIV [311]. Trachelogenin, a dibenzyl lignan, may be a novel inhibitor of hepatitis C because it blocks the penetration of viruses into hepatocytes, preventing the interaction of viruses with the CD81 host protein [312]. Other promising lignans with anti-HCV activity may be flax lignans, which inhibit viral replication [313]. Phillyrin has been shown to inhibit the expression of the influenza A virus nuclear protein (NP) gene and significantly block viral replication. Recent studies also highlight its potential against SARS-CoV-2, the virus behind COVID-19. Bioinformatics analysis revealed 192 shared targets and 25 pathways in co-infection with influenza and SARS-CoV-2, with HIF-1, PI3K-AKT, and RAS signaling pathways likely playing key roles in its antiviral action, offering new prospects for COVID-19 therapy [314]. Studies have shown that lignans from *Schisandra chinensis* possess antiviral properties. Schizandrin B and deoxyschizandrin effectively inhibit the DNA polymerase activity of HIV-1 reverse transcriptase, disrupting early stages of viral replication. Schizandrin A, on the other hand, suppresses dengue virus replication by activating the STAT1/2 signaling pathway and enhancing the interferon response. Schizandrin C stimulates the cGAS-STING pathway and promotes the production of interferon-β (IFN-β), leading to the inhibition of hepatitis B virus replication. Moreover, its combination with luteolin shows a synergistic antiviral effect in an HBV-infected mouse model [27]. The antiviral activity of lignans has been observed through cytopathic inhibition assays, syncytium formation assays by virus-infected cells, and inhibition of viral reverse transcriptase. The antiviral mechanisms of lignans include the inhibition of key viral enzymes (e.g., integrase, protease), disruption of viral fusion and internalization, as well as suppression of viral antigen expression and genome replication. This makes lignans promising candidates for further research into antiviral drug development. Xu et al. conducted a systematic review that included over 600 lignans evaluated for their antiviral activity [315]. Lignans, acting through various mechanisms, including membrane disruption and enzyme inhibition, exhibit promising antifungal activity against several human pathogenic fungi. Moreover, studies suggest that combining lignans with conventional antifungal drugs or natural antioxidants may produce synergistic effects, potentially reducing the required dosages and minimizing adverse effects [316,317]. According to the conducted studies, linseed extracts containing lignans exhibited moderate (between 70 and 90%) antifungal activity against *Aspergillus flavus* and *Aspergillus niger*, plant pathogens that can cause infections or allergic reactions in people with immune disorders. Additionally, the toxins secreted by these fungi are harmful to human health due to their potent hepatotoxic and carcinogenic properties [313]. The extract from *Larrea tridentata*, rich in two lignans: methylnordihydroguaiaretic acid and nordihydroguaiaretic acid—also demonstrated strong antifungal properties against *A.flavus* and *Aspergillus parasiticus* [318]. Antifungal activity of honokiol has been observed in studies on *Candida albicans*. The simultaneous addition of vitamin C may significantly enhance the antifungal effect of honokiol, whereas vitamin E reduces the effectiveness of honokiol against *C. albicans* [317]. One of the mechanisms of antifungal action of honokiol is that it acts as a prooxidant in *C. albicans*, causing mitochondrial dysfunction and increasing apoptosis of fungal cells [319]. Magnolol is another compound exhibiting antifungal activity against *C. albicans.* Studies have shown that magnolol inhibits yeast biofilm formation by 69.5%. Moreover, cell membrane damage, cell wall and plasma membrane ruptures, cell deformation and intracellular release were observed in magnolol-treated *C. albicans* cells [320]. Studies on other lignans, benzofuran derivatives, have shown that these compounds have antifungal activity, but this is often associated with toxic effects on mammalian cells [321]. Further pharmacokinetic and *in vivo* toxicity assessments are necessary to fully evaluate the therapeutic potential of lignans in treating human fungal infections.

In summary, lignans are promising compounds with multifaceted antimicrobial activity. Examples of lignans and microorganisms susceptible to their antimicrobial properties are presented in Table 8.

## 3. Summary

In summary, lignans—plant-derived polyphenols metabolized in the gut into estrogen-like enterolignans—exhibit a broad spectrum of biological activities. As presented in this review, their antioxidant, anti-inflammatory, hormone-modulating, and antimicrobial effects contribute to a wide range of health benefits. These include the prevention of hormone-dependent cancers, support for cardiovascular and metabolic functions, protection against neurodegenerative disorders, and the alleviation of menopausal symptoms. Such multifaceted actions position lignans as promising compounds in the prevention and management of chronic diseases, as well as valuable bioactive ingredients in functional foods.

Given their diverse benefits, lignans represent an important and expanding area of interest in both nutritional science and medical research. However, despite substantial evidence from in vitro and animal studies, clinical data remain limited. Further human studies are necessary to determine optimal dosing, improve understanding of their bioavailability, and confirm therapeutic efficacy. Future research should also address how individual factors—such as age, sex, and gut microbiota composition—influence lignan metabolism and activity. Additionally, the exploration of potential synergistic effects with other phytochemicals or pharmaceuticals may enhance their clinical utility. A particularly important direction for future investigation lies in evaluating the role of lignans in neurodegenerative diseases, metabolic syndrome, and hormone-dependent cancers through well-designed, large-scale clinical trials. Finally, the development of lignan-enriched functional foods or supplements with improved bioavailability could further facilitate their practical application in health promotion.

## Figures and Tables

**Table 1:** Lignans and their dietary sources, molecular formulas and structure.

Lignans	The Most Important Dietary Sources	Molecular Formula	Structure	References
Secoisolariciresinol	flaxseed, pumpkin seeds, sunflowers seeds, kiwi	C_20_H_26_O_6_	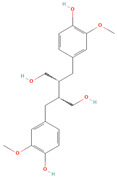	[1,2,3,4,5,6]
Secoisolariciresinol diglucoside (SDG)	flaxseed, sesame seeds	C_32_H_46_O_16_	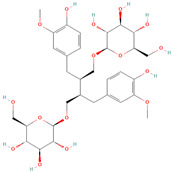	[7,8]
Matairesinol	flaxseed, sesame seeds, wine, oat, rye	C_20_H_22_O_6_	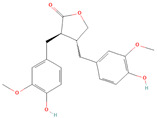	[8,9]
Lariciresinol	flaxseed, sesame seeds, sunflower seed, cashew, pumpkin seeds, buckwheat, barley, oat, rye, wheat, pineapple, apricot, strawberry, pear eggplant, curly kale, white cabbage, brussels sprout, garlic, French bean, sweet pepper, raisins, tomato paste	C_20_H_24_O_6_	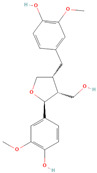	[3,5,8,9]
Pinoresinol	flaxseed, sesame seeds, buckwheat, oat, rye, curly kale, broccoli, white cabbage, brussels sprout, sauerkraut, garlic, apricot, strawberry, peach, nectarine, olive oil	C_20_H_22_O_6_	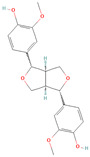	[5,9,10,11,12,13]
Pinoresinol diglucoside (PDG)	sesame seeds	C_32_H_42_O_16_	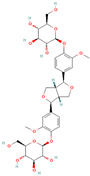	[14]
Arctigenin	burdock (*Arctium lappa*)—root, sprouts, seed infusion	C_21_H_24_O_6_	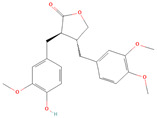	[15,16,17,18]
Arctiin	burdock (*Arctium lappa*)—seeds, leaves, fruits and roots	C_27_H_34_O_11_	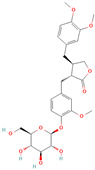	[8,19]
Hydroxymatairesinol	sesame seed, wheat, rye	C_20_H_22_O_7_	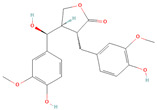	[8,20]
Medioresinol	flaxseed, sesame seed, rye, wheat, oat, lemons	C_21_H_24_O_7_	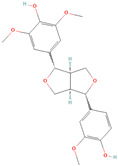	[8,9,12,21]
Sauchinone	roots of Asian lizard’s tail (*Saururus chinensis*)	C_20_H_20_O_6_	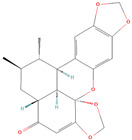	[22,23,24]
Sesamin	sesame seed and oil, wheat, rye	C_20_H_18_O_6_	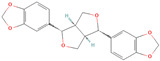	[2,9,25]
Sesamolin	sesame seed and oil	C_20_H_18_O_7_	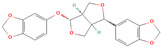	[7,8,25,26]
Syringaresinol	buckwheat, oat, rye, wheat, oranges, pineapple, sesame seed	C_22_H_26_O_8_	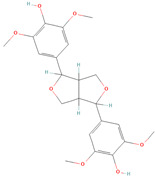	[8,9]
Schisandrin A	five-flavor fruit (*Schisandra chinensis*)	C_24_H_32_O_6_	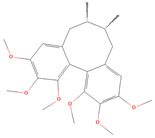	[27,28]
Schisandrin B	five-flavor fruit (*Schisandra chinensis*)	C_23_H_28_O_6_	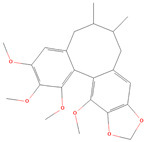	[28,29]
Kusunokinin	black pepper	C_21_H_22_O_6_	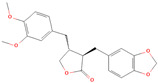	[30]
Honokiol	*Magnolia officinalis* whole plant (mostly bark)	C_18_H_18_O_2_	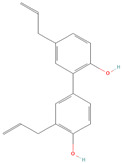	[31,32]
Podophyllotoxin	*Podophyllum peltatum* (Amierican mayapple), *Sinopodophyllum hexandrum* (Himalayan mayapple)	C_22_H_22_O_8_	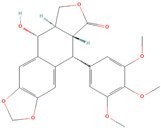	[33,34]
Macelignan	nutmeg mace of *Myristica fragrans*	C_20_H_24_O_4_	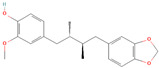	[35,36]
Tracheloside	Safflower (*Carthamus tinctorius*)—seeds; *Trachelospermi caulis*	C_27_H_34_O_12_	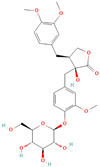	[37,38]

All 2D structures of the lignans presented herein were obtained from https://pubchem.ncbi.nlm.nih.gov accessed on 1 August 2025 Secoisolariciresinol PubChem Identifier: CID 65373 URL: https://pubchem.ncbi.nlm.nih.gov/compound/65373 accessed on 1 August 2025 Secoisolariciresinol diglucoside (SDG) PubChem Identifier: CID 9917980 URL: https://pubchem.ncbi.nlm.nih.gov/compound/Secoisolariciresinol-diglucoside accessed on 1 August 2025 Matairesinol PubChem Identifier: CID 119205 URL: https://pubchem.ncbi.nlm.nih.gov/compound/119205 accessed on 1 August 2025 Lariciresinol PubChem Identifier: CID 332427 URL: https://pubchem.ncbi.nlm.nih.gov/compound/332427 accessed on 1 August 2025 Pinoresinol PubChem Identifier: CID 73399 URL: https://pubchem.ncbi.nlm.nih.gov/compound/73399 accessed on 1 August 2025 Pinoresinol diglucoside (PDG) PubChem Identifier: CID 174003 URL: https://pubchem.ncbi.nlm.nih.gov/compound/174003 accessed on 1 August 2025 Arctigenin PubChem Identifier: CID 64981 URL: https://pubchem.ncbi.nlm.nih.gov/compound/64981 accessed on 1 August 2025 Arctiin PubChem Identifier: CID 100528 URL: https://pubchem.ncbi.nlm.nih.gov/compound/100528 accessed on 1 August 2025 Hydroxymatairesinol PubChem Identifier: CID 10948757 URL: https://pubchem.ncbi.nlm.nih.gov/compound/10948757 accessed on 1 August 2025 Medioresinol PubChem Identifier: CID 181681 URL: https://pubchem.ncbi.nlm.nih.gov/compound/181681 accessed on 1 August 2025 Sauchinone PubChem Identifier: CID 11725801 URL: https://pubchem.ncbi.nlm.nih.gov/compound/11725801 accessed on 1 August 2025 Sesamin PubChem Identifier: CID 72307 URL: https://pubchem.ncbi.nlm.nih.gov/compound/72307 accessed on 1 August 2025 Sesamolin PubChem Identifier: CID 101746 URL: https://pubchem.ncbi.nlm.nih.gov/compound/101746 accessed on 1 August 2025 Syringaresinol PubChem Identifier: CID 100067 URL: https://pubchem.ncbi.nlm.nih.gov/compound/100067 accessed on 1 August 2025 Schisandrin PubChem Identifier: CID 3001664 URL: https://pubchem.ncbi.nlm.nih.gov/compound/3001664 accessed on 1 August 2025 Schisandrin A PubChem Identifier: CID 155256 URL: https://pubchem.ncbi.nlm.nih.gov/compound/155256 accessed on 1 August 2025 Schisandrin B PubChem Identifier: CID 108130 URL: https://pubchem.ncbi.nlm.nih.gov/compound/108130 accessed on 1 August 2025 Kusunokinin PubChem Identifier: CID 384876 URL: https://pubchem.ncbi.nlm.nih.gov/compound/384876 accessed on 1 August 2025 Honokiol PubChem Identifier: CID 72303 URL: https://pubchem.ncbi.nlm.nih.gov/compound/72303 accessed on 1 August 2025 Podophyllotoxin PubChem Identifier: CID 10607 URL: https://pubchem.ncbi.nlm.nih.gov/compound/10607 accessed on 1 August 2025 Macelignan PubChem Identifier: CID 10404245 URL: https://pubchem.ncbi.nlm.nih.gov/compound/10404245 accessed on 1 August 2025 Tracheloside PubChem Identifier: CID 169511 URL: https://pubchem.ncbi.nlm.nih.gov/compound/Tracheloside accessed on 1 August 2025

**Table 2:** Protective mechanisms of lignans against inflammation and oxidative stress.

Lignan	Biological Activity	Mechanism of Action	Target Tissue/Protected Model	References
SDG	antioxidant	reduced oxidative damage by lowering Pb accumulation, restoring renal function, and enhancing enzymatic activity	rat model treated with lead acetate	[56]
		reduced ROS generation; increased FSHR expression, follicle count, mitochondrial DNA copy number, and slowed telomere shortening; improved nutrient metabolism in ovaries	ovaries in reproductive aging mice model	[57]
		reduced ROS and MDA; upregulated Nrf2/HO-1, SODs, and GPx-1; restored kidney morphology and antioxidant enzyme levels	kidneys of offspring from TFA-exposed mice	[58]
	anti-inflammatory	suppressed mRNA expression of inflammatory cytokines, improved intestal barrier integrity, ameliorated morphologic damage of the colon, modulated gut microbiota and short-chain fatty acids levels; effects depend partly on microbiota modulation	colonic inflammation caused by a common poor diet, high-fat diet	[59]
		reduced inflammatory cytokines levels in aortic tissue and plasma (IL-1β, IL-17A, TNF-α, MCP-1), inhibited inflammatory Mψs in atherosclerosis	aorta and vascular system of HFD-induced atherosclerosis mice	[60]
	antioxidant, anti-inflammatory	reduced oxidative stress, inflammation, and apoptosis via miR-101a/MKP-1-mediated inhibition of p38 and ERK signaling pathways	liver and kidney in BaP-treated mice	[61]
		inhibited IL-1β-induced inflammatory markers and ECM degradation via activation of Nrf2/HO-1 and inhibition of NF-κB pathway	cartilage degeneration (in vitro and in vivo)	[62]
Pinoresinol	antioxidant	activated Nrf2-mediated antioxidant response; reduced oxidative stress in human lung epithelial cells exposed to sodium arsenite-induced oxidative insults	human lung epithelial cells	[63]
	anti-inflammatory, antioxidant	decreased TNF-α, IL-6, IL-1β; suppressed LPS-induced ERK1/2 and p38 phosphorylation; reduced ROS generation in macrophages via MAPK pathway	LPS-stimulated Raw 264.7 macrophages	[64]
Pinoresinol diglucoside (PDG)	anti-inflammatory, antioxidant	decreased TNF-α, IL-1β, IL-6, NO, ROS, and MDA; increased SOD, GSH, GSH-Px; modulated NF-κB and activated Nrf2/HO-1 pathways	neuronal tissue in MCAO-induced brain ischemia/reperfusion injury in C57BL/6 mice	[65]
		inhibited TNF-α, IL-1β, ROS, and MDA; increased SOD and catalase; modulated TLR4/NF-κB and activated Nrf2/HO-1	neurons in Aβ1-42-induced Alzheimer’s disease model	[66]
Lariciresinol	anti-inflammatory, antioxidant	reduced inflammatory cytokines (TNF-α, IL-17), oxidative stress markers; inhibited NF-κB and TGF-β expression	CFA-induced rheumatoid arthritis in rats	[67]
Matairesinol	anti-inflammatory, antioxidant	inhibited microglial activation and pro-inflammatory cytokines; boosted SOD and GSH-Px; modulated MAPK, NF-κB, AMPK, Nrf2/HO-1 pathways	CLP-induced sepsis-mediated brain injury in rats	[68]
Arctigenin	anti-inflammatory, antioxidant	activated Nrf2/HO-1/NQO1; reduced NF-κB and ER stress markers (BiP, PERK, IRE1α, CHOP, caspase-12); lowered TNF-α, IL-1β, oxidative stress	cadmium-induced nephrotoxicity in rats	[69]
		reduced liver enzymes, suppressed MMP-2, restored glutathione, SOD, and glutathione reductase	CCl_4_-induced liver injury in rats	[70]
		reduced ROS and MDA levels, increased SOD activity, inhibited activation of NF-κB signaling, and activated the AMPK/SIRT1 antioxidant pathway	cardiomyocytes subjected to oxygen-glucose deprivation; myocardial tissue after acute myocardial ischemia/reperfusion	[71]
		attenuated bleomycin-induced pulmonary fibrosis by reducing ROS levels, increased SOD and GSH, and decreased MDA in lung tissue; inhibited collagen and α-SMA expression and modulated the TGF-β/p-Akt pathway	lung tissue in bleomycin-induced pulmonary fibrosis in mice	[72]
	antioxidant	reduced oxidative stress by decreasing lipid peroxidation and enhancing antioxidant enzymes (SOD, catalase, GSH)	streptozotocin-induced diabetic neuropathy in mice	[73]
	anti-inflammatory	suppressed TLR4-mediated NF-κB signaling by reducing interaction of AdipoR1 with TLR4 and CD14; inhibited production of proinflammatory cytokines; decreased APP, BACE1, and Aβ generation; prevented neuronal/synaptic injury and glial activation	neuroinflammation, neuronal injury, cognitive impairments in LPS-treated mice	[74]
		suppressed inflammation and NF-κB pathway activation in IL-1β-induced human nucleus pulposus cells by upregulating miR-483-3p; also inhibited apoptosis	cell model of intervertebral disc degeneration	[75]
Arctiin, Arctigenin	anti-inflammatory, antioxidant	inhibited TLR-4/Myd88/NF-κB and NLRP3 inflammasome; reduced ROS, TNF-α, IL-1β, TGF-β, α-SMA; regulated metabolic pathways and biomarkers	silica-induced pulmonary fibrosis (silicosis)	[76]
Arctiin	anti-inflammatory	reduced expression of TLR4 and NLRP3, inhibiting the inflammasome pathway; also downregulated STAT3 and TGF-β involved in tissue fibrosis and reduced cyclin D1 and CDK2	hippocampus in Alzheimer’s model rats	[77]
		inhibited glycolysis and inflammation via FGFR2/CSF1R signaling; reduced inflammatory cytokines and oxidative stress	liver tissue in high fat diet (HFD)-induced Non-alcoholic steatohepatitis (NASH)	[78]
		inhibited inflammation and pyroptosis via suppression of the TLR4/MyD88/NF-κB and NLRP3/Caspase-1/GSDMD pathways; reduced IgE, cytokines	skin integrity in DNCB-induced dermatitis	[79]
	antioxidant	activated AKT/NRF2/HO-1 signaling; reduced intracellular iron, reactive oxygen species, and lipid-ROS; restored mitochondria	chondrocytes in iron overload-induced knee osteoarthritis (KOA) advancement	[80]
	anti-inflammatory, antioxidant	inhibited MAPK pathway; reduced inflammatory cytokines (IL-1β, IL-6, TNF-α); improved oxidative stress markers (↑SOD, catalase, GPx; ↓ROS, MDA)	liver in high-fat diet-induced nonalcoholic fatty liver disease	[81]
		inhibited p38 and NF-κB activation; reduced inflammatory cell infiltration; corrected Th1/Th2 imbalance; increased superoxide dismutase (SOD) activity; reduced oxidative stress	lung tissue in ovalbumin-induced asthma	[82]
		activated Nrf2/HO-1 signaling; inhibited RIG-I/JNK MAPK pathway; reduced H9N2-induced proinflammatory cytokines (IL-6, TNF-α), COX-2, and PGE2; increased SOD2 and HO-1 expression	cells infected with H9N2 avian influenza virus	[83]
Honokiol	antioxidant	activated SIRT3/AMPK pathway; restored proliferation/apoptosis balance; improved antioxidant capacity in H_2_O_2_-induced ovarian follicles	ovarian tissue and small white follicles in aging chickens	[84]
		reduced oxidative stress (↓MDA, ↑GSH, ↑SOD), modulated IL-1β and TGF-β1 expression in kainic acid-induced neurodegeneration	hippocampus and cerebral cortex in rats	[55]
Medioresinol	anti-inflammatory, antioxidant	activated PI3K/AKT/mTOR signaling pathway; reduced oxidative stress and inflammation in myocardial ischemia-hypoxia model cells	H9c2 cardiomyocytes under oxygen-glucose deprivation	[21]
Sauchinone	antioxidant	restored mitochondrial function, reduced ROS production by improving electron transport in the electron transport chain; downregulated VAMP8	mitochondria in senescent cells	[85]
	anti-inflammatory, antioxidant	activated NRF2 signaling to reduce oxidative stress; inhibited NLRP3 inflammasome activation, reduced inflammation and apoptosis	heart tissue from Dox-induced injury	[86]
	anti-inflammatory	reduced inflammatory cytokines (TNF-α, IL-1β, IL-6); decreased inflammatory cell infiltration; restored colon tissue morphology	colon tissue integrity in DSS-induced ulcerative colitis (UC) model	[24]
Sesamin	anti-inflammatory, antioxidant	increased antioxidant enzymes (GSH, CAT, SOD); reduced MDA, IL-1β, TNF-α, and caspase-3; improved kidney function markers	kidney in rats (protection from cyclophosphamide-induced nephrotoxicity)	[87]
		reduced ROS, TNF-α, IL-1β, and inflammatory cell recruitment; inhibited HMGB1/TLR4/NF-κB signaling pathway	liver in mice (protection from acetaminophen-induced acute liver injury)	[88]
		inhibited Ang-II-induced oxidative stress, apoptosis, and inflammation in H9c2 cells; reduced ROS, NADPH oxidase activity, and hypertrophic markers (ANP, BNP, β-MHC)	cardiomyocytes (H9c2 cells), heart function	[89]
		decreased ROS and NO levels; modulated oxidative and inflammatory gene expression; showed no embryotoxicity or cardiotoxicity	zebrafish embryos (oxidative and inflammatory stress)	[90]
Sesamol	anti-inflammatory, antioxidant	inhibited CYP2E1 and NOX2 activity; suppressed NF-κB activation and TNF-α expression; enhanced Nrf2 transcription and upregulated HO-1 and NQO1, reducing oxidative stress and inflammation	liver in HFD-induced hepatic steatosis	[91]
Sesamin, Sesamol	antioxidant	reduced H_2_O_2_-induced ROS and apoptosis in SH-SY5Y cells by activating SIRT1–SIRT3–FOXO3a signaling, decreasing BAX, and increasing BCL-2 expression	human neuroblastoma (SH-SY5Y) cells	[92]
Sesamin, Sesamolin	antioxidant	decreased ROS production; reduced oxidative stress-induced apoptosis; normalized ERK1/2 activation	neural cells (PC12 cells) in Parkinson’s disease model	[93]
Sesamin, Sesamolin, Sesamol	antioxidant	reduced TG/TC levels and oxidative stress in steatosis HepG2 cells; activated AMPK and PPAR pathways to promote fatty acid oxidation and reduce lipogenesis	hepatic lipid metabolism, liver cells	[94]
Syringaresinol	antioxidant	exhibited strong radical scavenging activity (DPPH and ABTS assays); reduced expression of MMP-2 and MMP-9 via upregulating autophagy (LC3B); antioxidant effect decreased ROS-induced ECM degradation	human keratinocytes (HaCaT cells) under H_2_O_2_-induced oxidative stress (skin aging model)	[95]
		decreased intracellular ROS; enhanced Nrf2 antioxidant pathway and related enzymes; reduced DNA damage (lower CPD photoproducts); lowered senescence markers (MMPs, p21); inhibited MAPKs phosphorylation and NF-κB	human epidermal keratinocytes (HEKs) under UVB irradiation (photoaging model)	[96]
	anti-inflammatory	activates ER/SIRT1; inhibits NLRP3 inflammasome and pyroptosis in cardiomyocytes	cardiac function and myocardial tissue in sepsis-induced cardiac dysfunction (mice and cardiomyocytes)	[50]
		inhibits NF-κB pathway; reduces IL-6, TNF-α, MMP-13, NO, PGE2; protects ECM	cartilage and joint tissue in osteoarthritis (mouse model and chondrocytes)	[51]
		enhances intestinal barrier; reduces TNF-α, IL-6, IFN-γ, COX-2; regulates PI3K-Akt/MAPK/Wnt pathways	intestinal epithelial barrier and colon tissue (in UC mice and Caco-2 cells)	[52]
		inhibited NLRP3 inflammasome activation and pyroptosis via estrogen receptor-β pathway; reduced cytokines, MPO, M1 macrophages	lung tissue in CLP-induced acute lung injury mice and RAW264.7 cells	[97]
	anti-inflammatory, antioxidant	activates Nrf2 antioxidant pathway; downregulates HIF-1α/VEGF pathway; reduces oxidative stress and inflammation	retinal tissue in diabetic mice and endothelial cells under high glucose	[49]
		upregulates NRF2; inhibits NLRP3/Caspase-1/GSDMD pyroptosis pathway	renal structure and function in diabetic nephropathy (STZ-induced diabetic mice)	[98]
Schisandrin A	antioxidant	reduced oxidative stress and inhibited PKCβ expression, leading to downregulation of fibrosis markers	kidney tissue in Unilateral Ureteral Obstruction mice model and cell lines	[54]
Schisandrin B	antioxidant	inhibited oxidative stress and ferroptosis via upregulating GPX4 and reducing ROS in THP-treated rats	liver in pirarubicin (THP)-induced hepatotoxicity	[53]

↑ upregulation, ↓ downregulation.

**Table 3:** Neuroprotective effects of lignans in neurodegenerative disease models.

Lignan	Disease	Mechanism of Action	Model/System	References
SDG	Alzheimer’s disease	improved spatial, recognition, and working memory. Enhanced CREB/BDNF and PSD-95 expression, reduced β-amyloid deposition and levels of TNF-α, IL-6, and IL-10. Altered gut microbiota composition, increased serum levels of END and ENL. Correlation analysis linked END and ENL to cognitive performance and neuroinflammation. GPER was identified as a mediator of anti-inflammatory responses	female AD mice model	[117]
7-hydroxymatairesinol	Parkinson’s disease	improved motor function and slowed dopaminergic terminal loss	6-OHDA PD rat model	[112]
Honokiol	Alzheimer’s disease	increased SIRT3 expression and activity, improved ATP production, and reduced mitochondrial ROS. Restored AβO-induced mitochondrial dysfunction and rescued memory deficits in early AD stages	PS1^V97L AD mice model	[114]
		improved memory performance in the Morris Water Maze. Reduced hippocampal apoptosis, ROS production, and mitochondrial dysfunction. Inhibited NF-κB activation, APP, and β-secretase expression	AD mice model	[118]
		improved cognition, reduced Aβ_1_–_42_ deposition, promoted neuron survival via SIRT3-mediated mitochondrial autophagy; effects blocked by 3-TYP/CsA	AD mice & hippocampal neuronal cell model	[113]
	Alzheimer’s disease	reduced microglial senescence and inflammation. Decreased ROS, NF-κB, p21, γ-H2AX, and SASP markers. Increased IL-10. Inhibited Notch signaling Via Jagged1 downregulation	BV2 microglia cells in vitro	[119]
		enhanced survival and growth of iPSC-derived neurons	human AD iPSC-derived neurons	[120]
		improved spatial memory and retention, restored acetylcholine, GABA, and glutamate levels, reduced NF-κB and Aβ(1–42) expression, and protected against neuronal damage, indicating antioxidant, anti-inflammatory, and neuroprotective effects	ICV-STZ-induced AD rats	[121]
	Amyotrophic lateral sclerosis	improved motor neuron viability, enhanced GSH synthesis and NRF2-ARE signaling, restored mitochondrial dynamics, extended lifespan and motor function in ALS mice	SOD1-G93A ALS cell model & transgenic mice	[116]
Macelignan	Alzheimer’s disease	reduced phosphorylation of Tau at Thr231, Ser396, and Ser404 in overexpressing cell lines, and at Ser404 in mouse primary neural cells. Increased autophagy and enhanced PP2A activity to regulate Tau phosphorylation. Activated the PERK/eIF2α signaling pathway, leading to reduced BACE1 translation, inhibited APP cleavage, and suppressed Aβ deposition	Tau-overexpressing cell lines, N2a/SweAPP cells	[122]
Pinoresinol diglucoside (PDG)	Alzheimer’s disease	reduced proinflammatory cytokines, oxidative stress, and neuronal apoptosis. Modulated TLR4/NF-κB and Nrf2/HO-1 pathways	AD mice model	[66]
Pinoresinol	Alzheimer’s disease	improved memory and restored long-term potentiation Via calcium-permeable AMPA receptors and Akt signaling. Reduced neuroinflammation and synaptic deficits	AD mice model	[123]
(–)-7-epi-Pinoresinol, (+)-Medioresinol, (+)-Diapinoresinol	Parkinson’s disease	increased cell viability and antioxidant enzyme activity (SOD, GPx), reduced ROS and LDH levels, and activated the PI3K/Akt/GSK-3β/Nrf2 pathway	PC-12 cells induced by H_2_O_2_ (PD model)	[124]
Arctiin	Alzheimer’s disease	improved hippocampal structure and behavioral performance. Reduced expression of TLR4 and NLRP3, thereby inhibiting the inflammasome pathway. Also regulated STAT3 and TGF-β, contributing to reduced tissue fibrosis, and inhibited cell cycle proteins cyclin D1 and CDK2	AD rat model	[77]
Arctigenin	Alzheimer’s disease	improved memory and reduced tau phosphorylation in the hippocampus and neuroinflammation in the cortex. modulated mitochondrial function Via tricarboxylic acid cycle and electron transport proteins	AD mice model	[125]
	neurodegeneration-related diseases	improved memory and reduced Aβ, APP, and BACE1 levels. Inhibited TLR4/NF-κB signaling, glial activation, and proinflammatory cytokines Via AdipoR1–TLR4 interaction	LPS-treated mice & BV2 cells	[74]
	multiple sclerosis	reduced calcium influx, neuronal hyperactivity, and excitotoxicity in cortex at preclinical stage; restored neural communication during remission stage	EAE mouse model (MS)	[126]
Sesamin	Alzheimer’s disease	prevented impairment of long-term potentiation at perforant path–dentate gyrus synapses; increased excitatory postsynaptic potential slope and population spike amplitude; reduced oxidative stress	AD rat model	[127]
		reduced advanced glycation end product-induced microglial inflammation by suppressing NF-κB, p38, JNK pathways and downregulating RAGE expression	BV2 microglial cells in vitro	[128]
Sesamin, Sesamolin	Parkinson’s disease	reduced reactive oxygen species and apoptosis, increased survivin, and normalized ERK1/2 signaling	6-OHDA-induced PC12 cells (PD model)	[93]
Schisandrin	Alzheimer’s disease	improved mitochondrial membrane potential, ATP production, cytochrome c oxidase activity, biogenesis, and fusion–fission balance in Aβ1–42-treated cells	rat hippocampal neurons + Aβ1–42	[129]
		improved cognition in AD mice by reducing Aβ levels, inhibited neuronal apoptosis and NLRP1 inflammasome-mediated pyroptosis in hippocampal neurons	AD mice model	[130]
		improved cognitive impairment and hippocampal cell loss; modulated gut microbiota composition and corrected metabolic imbalances in feces, plasma, and brain, particularly involving bile acid biosynthesis and lipid-related pathways	AD rat model	[131]
Schisandrin A	Alzheimer’s disease	improved cell viability, reduced apoptosis, oxidative stress, and inflammation; effects were linked to ERK/MAPK pathway activation	SH-SY5Y and SK-N-SH cells (Aβ_(25–35)_ model)	[115]
	Parkinson’s disease	improved motor behavior and dopaminergic neuron survival; reduced IL-6, IL-1β, TNF-α; increased SOD and autophagy-related proteins (LC3-II, beclin1, parkin, PINK1); mTOR upregulation	MPTP-induced PD mouse model	[111]
Schisandrin B	Alzheimer’s disease	Inhibited neuronal ferroptosis Via GSK3β/Nrf2/GPX4 and FSP1 pathways, reduced TNF-α release, and prevented M1 microglia activation, improved cognition and pathology	3 × Tg AD mice; SH-SY5Y/APP695swe cells	[132]
Schisandrin B (as M@Sch B micelles)	Alzheimer’s disease	inhibited Aβ aggregation, reduced ROS, enhanced antioxidant enzyme activity, and regulated aging-related genes, improved lifespan, mobility, and delayed AD-like symptoms in C. elegans	*C. elegans* (CL4176)	[133]
*Schisandra chinensis* lignans	Alzheimer’s disease	improved cognition and reduced p-Tau and neuronal loss. Inhibited oxidative stress and ferroptosis Via Nrf2/FPN1 signaling. Increased FPN1, SLC7A11, GPX4; decreased TFR, DMT1, FACL4	AD mice model, Erastin-treated HT22 cells	[134]
	Parkinson’s disease	reduced motor deficits and dopaminergic neuron loss Via suppression of TRPV1-AMPK-NLRP3 signaling, autophagy induction, and neuroinflammation control	MPTP-induced PD mice; LPS-activated BV2 cells	[135]
Matairesinol, SECO, Arctigenin, Arctiin	Alzheimer’s disease	inhibited NO production dose-dependently. Matairesinol and SDG reduced NO by 60% and downregulated iNOS and COX-2 expression. Potential for treating neuroinflammation	LPS-stimulated microglia	[136]

**Table 4:** Therapeutic Effects of Plant-Derived Lignans in Osteoporosis.

Lignan	Key Findings	Model/System	References
SDG	SDG improved bone microarchitecture, reduced inflammation, and increased bone formation markers Via estrogen receptor (ERα/ERβ) modulation	Ovariectomized (OVX) rat model	[148]
SDG from flaxseed (administered Via fermented milk)	Significant improvements in bone mineral density (~12–16% BMD increase after 8 weeks) and trabecular thickness; decreased trabecular separation	OVX rat model	[149]
SDG, tracheloside (TCL)	SDG and TCL showed strong, dose-dependent anti-osteoporotic effects, significantly upregulating osteogenic genes (Runx2, SP7, OPG, etc.)	alloxan-induced zebrafish model	[150]
Pinoresinol diglucoside (PDG)	PDG potentially acts Via PI3K-Akt and estrogen signaling pathways, targeting genes like BCL2, IL6, MARK3, suggesting multi-target mechanisms in osteoporosis prevention	Rat model (in vivo metabolism study + network pharmacology)	[151]
	PDG improved bone mineralization, corrected spinal and cartilage defects, and enhanced motor function. PDG upregulated the Wnt signaling pathway	Dexamethasone-induced zebrafish model	[152]
	PDG inhibited osteoclast differentiation and bone resorption by suppressing NF-κB and AKT pathways Via PTEN stabilization. It reduced formation of F-actin rings, downregulated NFATc1, c-Fos, CTSK, TRAP, and blocked RANKL/RANK signaling, especially in early-stage osteoclast development	RANKL-induced osteoclastogenesis in RAW264.7 cells	[153]
Arctiin (ARC)	ARC restored osteoblast viability, reduced apoptosis, and promoted mineralization. It acted through the PI3K/Akt pathway to enhance osteogenesis and cell survival, while also reducing oxidative stress. In vivo, ARC improved bone microarchitecture and biochemical markers, supporting its use in iron overload–induced osteoporosis	In vitro (MC3T3-E1 osteoblast cell line) + in vivo (iron-overload mice)	[147]
Arctigenin	Arctigenin suppressed adipogenesis and enhanced osteogenesis in BMSCs by reducing lipid droplet formation and downregulating lipogenic proteins. In OVX rats, it reduced bone loss, improved lipid metabolism, and promoted bone formation. Mechanistically, it acted Via the MEK1/PPARγ/β-catenin pathway, blocking PPARγ–β-catenin interaction and promoting nuclear β-catenin accumulation	In vitro (bone marrow mesenchymal stem cells (BMSCs) from OVX rats); In vivo (OVX rat model)	[154]
Sesamin	Sesamin activated BMP2 signaling and enhanced angiogenesis and chondrogenesis in vitro. This stimulatory effect was eliminated when an ERα inhibitor was applied. In OVX mice, it improved callus formation, enlarged cartilaginous and callus area, and accelerated fracture healing	In vitro (BMSCs, HUVECs); In vivo (OVX fracture mice)	[155]
	Sesamin promoted osteogenesis Via Wnt/β-catenin and inhibited osteoclastogenesis via NF-κB suppression. It regulated bone remodeling in a DANCR-dependent manner, reversing OVX-induced bone loss and reducing elevated serum DANCR levels. Suggests therapeutic potential for osteoporosis patients with high DANCR expression.	In vitro (BMSCs); In vivo (OVX fracture mice)	[146]
Lignan-rich fraction from *Sambucus williamsii*	Bone protection was mediated through gut microbiota modulation and serotonin suppression, leading to enhanced bone formation	OVX rat model	[156]
Matairesinol, secoisolariciresinol, pinoresinol, lariciresinol (dietary intake)	Highest quartile of total lignan intake was associated with a 76% lower risk of hip fracture, individual lignans with up to 62% reduction	Epidemiological study (elderly Chinese adults, hip fracture cases)	[157]

**Table 5:** Mechanisms of cardioprotective action of lignans.

Lignans	Disease	Action	Mechanism	**References**
Secoisolariciresinol diglucoside (SDG)	atherosclerosis	control of cholesterolemic status and improvement of dyslipidemia and redox state	regulation of the apelin/APJ signaling pathway	[165]
		inhibition of inflammation and modulation of gut homeostasis	regulation of macrophages, Treg cells, and γδT cells	[60]
		inhibition of inflammation and apoptosis	inhibition of the Akt/IκB/NF-κB pathway	[166]
	myocardial ischemia/reperfusion injury	an increase in capillary and arteriolar density along with enhanced left ventricular function	upregulation of HO-1, VEGF, and p-eNOS expression	[167]
		reduction in infarct size and cardiomyocyte apoptosis; increased capillary density and improved myocardial function	upregulation of VEGF, Ang-1, and p-eNOS protein expression	[168]
	cardiac hypertrophy	marked reduction in cardiac oxidative stress, inflammation, and apoptosis	suppression of upregulated ER stress markers GRP78, PERK, ATF-4, CHOP, NF-κB, and SREBP1c expression	[169]
	ischemic heart disease	inhibition of apoptosis	activation of the JAK2/STAT3 signaling pathway	[170]
Matairesinol	cardiac hypertrophy	alleviation of cardiac hypertrophy and fibrosis, preservation of cardiac function, and marked reduction in cardiomyocyte apoptosis and oxidative damage	upregulation of Prdx1 expression and inhibition of the PI3K/Akt/FoxO1 pathway	[171]
Pinoresinol	cardiac hypertrophy	prevention of cardiac histomorphological damage, reduction in hypertrophic biomarker upregulation, and attenuation of fibrosis and inflammation	inhibition of the AKT/mTOR/NF-κB signaling pathway activation	[172]
Pinoresinol diglucoside	myocardial ischemia	amelioration of H/R-induced cardiomyocyte injury	regulation of miR-142-3p and HIF1AN	[173]
	heart failure	inhibition of myocyte fibrosis, apoptosis, oxidative stress, and inflammation in pressure overload-induced cardiac injury	regulation of AMPK/SIRT3/RIG-1 signaling pathway	[174]
Arctigenin	myocardial ischemia/reperfusion injury	reduction in apoptosis, inflammation, and oxidative stress	enhancement of the AMPK/SIRT1 pathway and repression of NF-κB pathway activation.	[71,175]
	myocardial infarction	exhibition of antioxidative and anti-inflammatory effects	regulation of iNOS, COX-2, ERK1/2 and HO-1	[108]
		improvement of cardiac injury after MI through reduction in infarct size, enhancement of heart function, and inhibition of cardiac cell death	modulation of macrophage polarization Via the NFAT5-induced signaling pathway	[176]
	hypertension	reduction in systolic blood pressure and amelioration of endothelial dysfunction	enhancement of eNOS phosphorylation and reduction in NADPH oxidase-mediated superoxide anion generation	[177]
Medioresinol	myocardial infarction	reduction in oxidative stress and inflammatory responses	activation of the PI3K/AKT/mTOR pathway	[21]
Sauchinone	doxorubicin (dox)-induced cardiomyopathy (dic)	alleviates Dox-induced chronic cardiac injury but also significantly delays the progression of acute DIC	inactivation of the NLRP3 inflammasome and NRF2-mediated antioxidant pathways	[86]
	hypertension	inhibition of angiotensin II-induced proliferation and migration of vascular smooth muscle cells	inhibition of Ang II-induced over-activation of the TGF-β1/Smad3 signaling pathway	[178]
	myocardial ischemia/reperfusion injury	reduction of the infarct size	inhibition of phosphorylation of p38 and JNK death signaling pathways	[179]
		enhancement of antioxidant capacity and suppression of cardiac myocyte apoptosis	activation of Akt/eNOS Signaling Pathway	[180]
Sesamin	myocardial infarction	marked reduction in myocardial apoptosis in the border zone; reduction of myocardial apoptosis and inflammatory response.	downregulation of cytokine expression, inactivation of NF-κB signaling, and reduction in p-JNK protein levels.	[161]
	coronary heart disease	improvement of lipid metabolism and vascular endothelial function, inhibition of myocardial cell apoptosis	reduction in Caspase-12 and ICAM-1 protein expression, associated with activation of the PI3K-Akt-eNOS signaling pathway.	[181]
	myocardial hypertrophy	inhibition of oxidative stress, apoptosis, and inflammation	reduction in Ang II–induced increases in ANP, BNP, and β-MHC expression, elevation of NADPH oxidase activity and ROS production, and suppression of superoxide dismutase (SOD) activity	[89]
Syringaresinol	myocardial infarction	amelioration of MI-induced cardiac dysfunction, reduction in infarct size, and attenuation of myocardial hypertrophy, fibrosis, inflammation, and apoptosis	partial reversal of AKT1, EGFR, CASP3, SRC, NFKB1, HSP90AA1, HIF1A, MMP9, and ESR1 expression	[182]
Schisandrin A	chronic heart failure	ameliorated myocardial hypertrophy	inhibition of the expression levels of atrial natriuretic peptide, B-type natriuretic peptide, B-myosin heavy chain and blocked AKT/CREB activation Via miR-155	[183]
Schisandrin B	atrial fibrillation	protects against Ang II-induced ferroptosis, atrial fibrosis, and atrial fibrillation	activation of the SIRT1 pathway	[184]
	myocardial infarction	reducetion of inflammation, inhibition of apoptosis, and improvement of cardiac function after ischemic injury	down-regulation of some inflammatory cytokines, activation of eNOS pathway	[185]
	myocardial ischemia/reperfusion injury	reduction in the apoptotic index and serum markers of myocardial infarction	the PI3K/Akt signaling pathway (Upregulation of phosphorylated Akt expression, along with a reduction in the Bcl-2-like protein 4/Bcl-2 ratio and cleaved caspase-3 expression)	[162]
		reductions in infarct volume, neurological score, apoptotic neuron count, and levels of inflammatory signaling molecules	inhibition of TLR4/NF-κB signaling pathway	[186]
		reduction in myocardial infarct size, enhancement of antioxidant capacity, and attenuation of ER stress-induced apoptosis	decreasing oxidative reaction, suppressing ATF6 and PERK pathway, and attenuating ER stress-induced apoptosis	[187]
	heart failure	improve pathological myocardial remodeling and cardiac function induced by pressure overload	inhibition of the MAPK signaling pathway	[188]
	vascular endothelial dysfunction	amelioration of oxidative stress, mitochondrial membrane-potential depolarization and apoptosis in angiotensin II-challenged rat aortic endothelial cells	inhibition of Keap1 and activation of Nrf2 pathway, promotion the expression of downstream antioxidant genes Ho1 and Nqo1	[189]
	vascular remodeling	inhibition of inflammation and oxidative stress	suppressing NF-κB activation	[190]
	myocardial fibrosis	prevent Ang II-infused cardiac fibrosis	regulates the SIRT1/PI3K/Akt pathway	[191]
	myocardial inflammation	protect against myocardial inflammatory injury and tissue remodeling	inhibition of MyD88 signaling, increases in the production of inflammatory cytokines and expression of remodeling genes	[192]
Schisandrin C	atherosclerosis	ox-LDL degradation	regulation the autophagy pathway mediated by PI3K/AKT/mTOR	[193]
		inhibited proliferation and migration, attenuated lipid accumulation, reduced foam cell formation, suppressed inflammation in VSMCs	arresting cell cycle and targeting JAK2 to regulating the JAK2/STAT3 pathway	[194]
Schisandrin	heart failure	reduction in cardiomyocyte apoptosis rate, increase in the cell surface area-to-protein/DNA ratio, and elevation of mitochondrial membrane potential	reduction in JAK2 and STAT3 expression and significant reduction in the BAX/Bcl-2 ratio	[195]
Honokiol	post-myocardial infarction heart failure	reduced the abnormality of mitochondrial membrane potential (MMP) and apoptosis of cardiomyocytes	Ucp3-Mediated Reactive Oxygen Species Inhibition	[196]
		enhancing autophagic flux and reducing intracellular ROS production	enhanced autophagic flux is associated with the Akt signaling pathway	[197]
	hypertension	meliorates hypertension and endothelial dysfunction	inhibiting HDAC6-mediated cystathionine γ-lyase degradation	[198]
		significantly reduced blood pressure	inhibiting renal CYP4A and soluble epoxide hydrolase (sEH), reducing vasoconstrictive 20-HETE)	[199]
	myocardial ischemia/reperfusion injury (mi/ri)	suppressing mitochondrial apoptosis	reduce the MI/RI-induced cTnT and CK-MB levels, apoptosis index, and mitochondrial swelling in cardiomyocytes Via activating the PI3K/AKT signaling pathway	[200]
	cardiac hypertrophy	protected against myocardial hypertrophy, fibrosis and dysfunction	promoting dissociation of the Nur77–LKB1 complex and activating the AMPK pathway	[201]
	atherosclerosis	inhibits carotid artery atherosclerotic plaque formation	Inhibition of the inflammatory response, oxidative stress, excessive production of NO, and the activation of NF-κB signaling pathway	[202]
		suppresses migration and matrix metalloproteinase (MMP) expression	blocking NF-κB activation Via the ERK signaling pathway	[203]

**Table 6:** Mechanisms of action of selected lignans in the treatment and prevention of diabetes mellitus.

Lignans	Disease	Mechanism	References
SDG	type 2 diabetes mellitus	suppression of the expression of phosphoenolpyruvate carboxykinase (PEPCK) gene	[227]
	diabetes mellitus	inhibition of ROS level mediated increased level of enzymatic and non-enzymatic antioxidants	[228]
Matairesinol	type 2 diabetes mellitus	reduction in blood glucose and plasma insulin and improvement in the level of hepatic enzymes Via decreasing hepatocyte apoptosis via inhibition of DPP-4	[229]
Lariciresinol	type 2 diabetes mellitus	inhibition of α-glucosidase activity; stimulation of glucose uptake by enhancing the translocation of GLUT4 and glycogen content Via activating the insulin signaling pathway (e.g., IRS-1 and Akt signaling)	[230]
Arctigenin	type 2 diabetes mellitus	protected from insulin resistance; inhibition of toll-like receptor 4 inflammatory signaling to reactivate IRS-2/GLUT4	[231]
	diabetic nephropathy	reduction in NF-κB p65 phosphorylation likely through activation of PP2A	[232]
		exerted antioxidant and antiapoptotic effect with concomitant activation of autophagy and downregulation of AKT/mTOR pathway	[73]
Syringaresinol	diabetic nephropathy	inhibition of the NLRP3/Caspase-1/GSDMD pyroptosis pathway by upregulating NRF2 signaling	[98]
		downregulating HIF-1α/VEGF Via Activating Nrf2 Antioxidant Pathway	[52]
Syringaresinol	diabetic cardiomyopathy	suppression of antioxidant kelch-like ECH-associated protein 1 (Keap1)/nuclear factor-E2-related factor 2 (Nrf2) system and abnormal activation of transforming growth factor-β (TGF-β)/mothers against decapentaplegic homolog (Smad) signaling pathway	[233]
Syringaresinol-di-O-β-D-glucoside (SOG)	type 2 diabetes mellitus, diabetic nephropathy	decreasing the levels of oxidative stress through downregulation of the expression of NT and TGF-β1 in kidneys	[234]
Schisandrin B	type 2 diabetes mellitus	stimulation of insulin secretion through GLP-1R/cAMP/PKA signaling pathway	[235]
	diabetic cardiomyopathy	affection of MyD88 and inhibition of MyD88-dependent inflammation	[236]
Schisandrin	diabetic nephropathy	inhibit inflammation through PI3K/Akt and NF-κB signaling pathways; inhibit TGF-β1-induced renal fibrosis	[237]
Honokiol	type 2 diabetes mellitus	inhibition of hepatic CYP2E1 activity	[238]
		amelioration of hepatic steatosis by inhibiting hepatic lipogenic enzymes activity; improvement of hepatic inflammation, as shown by the decrease in TNF-α and IL-6 expression; anti-diabetic and anti-adiposity effects related to the inhibition of gluconeogenic enzymes and their mRNA expression	[239]
		reduction in oxidative stress and insulin resistance by activating SIRT3	[240]
		improvement in the insulin sensitivity by targeting PTP1B	[241]
	type 1 diabetes mellitus	reduction in oxidative stress and apoptosis through activating the SIRT1-Nrf2 signaling pathway	[242]

**Table 8:** Examples of lignans and target microbes demonstrating sensitivity to their antimicrobial properties.

Lignan/Plant	Pathogen	Reference
Alyterinates/*Alyxia schlechteri*	*Pythium insidiosum*	[322]
Cinaguaiacin, Demethoxyisoguaiacin/*Artemisia cina*	*Staphylococcus aureus*, *Listeria monocytogenes*, *Escherichia coli*, *Pseudomonas aeruginosa*, *Bacillus cereus*, *Klebsiella pneumoniae*, *Pasteurella multocida*, *Salmonella enterica*	[309]
Cycloolivil, Ferruginan/*Olea ferruginea*	*E. coli*, *K. pneumoniae*, *Seratiam marcescens*, *Citrobacter freundii*, *Vibrio vulnificus*, *Enterobacter aerogenes.*	[323]
Hinokinin/*Commiphora leptophloeos*	*S. aureus*, *S. aureus* (*MRSA*)	[302]
Honokiol, Magnolol	*S. aureus* (*MRSA*), *Candida albicans*	[317,320,324]
7-hydroxymatairesinol	*Staphylococcus epidermidis*, *C. albicans*, *Proteus* spp., and *Klebsiella spp.*	[306]
8-hydroxypinoresinol/*Strombosia grandifolia*	*S. aureus*, *Streptococcus pneumoniae*, *E. coli*, *Salmonella typhi*, *C. albicans*	[325]
Justicidin B	vesicular stomatitis virus, *Sindbis virus*	[326,327]
Lignanas from *Euclea natalensis*	bacteria that cause periodontal disease and caries	[328]
Lignans from *Forsythia viridissima*	coxsackievirus B3, human rhinovirus 1B	[329]
Methylnordihydroguaiaretic acid, Nordihydroguaiaretic acid	*Aspergillus. flavus* and *Aspergillus parasiticus*.	[318]
Nortrachelogenin	*E. coli*, *L. monocytogenes.*	[301,310]
Phyllanins/*Phyllanthodendron dunnianum*	*S. aureus MRSA*, *Enterococcus faecalis*, *P. aeruginosa* and *E. coli.*	[330]
Phillyrin/*Forsythia suspensa (Thunb.) Vahl*	*P. aeruginosa*, *K. pneumonia*, *E.coli*, *influenza A virus*, *coronavirus*	[314]
Schisandrins, deoxyschizandrin and other lignan/*Schisandra chinensis*	*S.s aureus*, *L. monocytogenes*, *B. subtilis*, *B. cereus*, *Salmonella enterica subsp. enterica serovar Typhimurium*, *P. aeruginosa*, *human immunodeficiency virus 1 Enterobacter aerogenes and E. coli*, *hepatitis B virus*, *dengue virus*	[27]
SDG and other flax lignans/*Linum usitatissimum*	*S. aureus*, *E. coli*, *P. aeruginosa*, *B. subtilis*, *C. albicans*, *Hepatitis C Virus*, *A. flavus*, *Aspergillus niger*	[304,305,313]
Sesamin	*E. coli*	[303]
Silymarin and derivatives	hepatitis Cand B virus, dengue virus, Chikungunya virus, Mayaro virus, influenza virus, human immunodeficiency virus	[331]
Styraksjaponozyd C/*Styrax japonica*	*C. albicans*	[332]
Trachelogenin	hepatitis C virus	[312]

## Data Availability

No new data were created or analyzed in this study.
